# Effects of Different Postharvest Precooling Treatments on Cold-Storage Quality of Yellow Peach (*Amygdalus persica*)

**DOI:** 10.3390/plants11182334

**Published:** 2022-09-06

**Authors:** Yuchen Zhang, Meijie Guo, Jun Mei, Jing Xie

**Affiliations:** 1 College of Food Science and Technology, Shanghai Ocean University, Shanghai 201306, China; 2 National Experimental Teaching Demonstration Center for Food Science and Engineering, Shanghai Ocean University, Shanghai 201306, China; 3 Shanghai Professional Technology Service Platform on Cold Chain Equipment Performance and Energy Saving Evaluation, Shanghai 201306, China

**Keywords:** yellow peaches, precooling, antioxidant enzymes, postharvest quality, shelf life

## Abstract

The rapid precooling of yellow peaches after harvest can minimize the tissue damage and quality deterioration of yellow peaches during postharvest storage. Refrigerator precooling (RPC), cold-water precooling (CWPC), strong-wind precooling (SWPC), fluidized-ice precooling (FIPC), and vacuum precooling (VPC) were used to precool the fresh yellow peaches. The yellow peaches after different precooling treatments were stored at 4 °C for 15 days. CWPC and RPC can effectively retard the respiration and ethylene peak production, reduce the quality loss of yellow peaches during postharvest storage, maintain the color and fruit hardness of yellow peaches, inhibit browning, maintain the contents of soluble solids, titratable acids, and ascorbic acid, increase the activity contents of superoxide dismutase (SOD) and peroxidase (POD), inhibit the decrease in the phenylalanine ammonia-lyase (PAL) activity, and delay the increase in the polyphenol oxidase (PPO) activity. The shelf life of yellow peaches with cold-water precooling and refrigerator precooling reached 15 days, which was 6 days longer than those of the VPC- and FIPC-treated samples, and 3 days longer than that of the SWPC-treated samples. Therefore, CWPC and RPC were effective methods to prolong the storage period and maintain the quality of yellow peaches during postharvest storage.

## 1. Introduction

Yellow peaches are an important economic fruit in China and some Mediterranean countries, and they are a nutritious and sweet-tasting fruit [1]. Yellow peaches are rich in vitamins, phenolic compounds, and various trace elements, while their sweet and crunchy taste and pleasant aroma increase consumer interest. However, yellow peaches breathe vigorously during postharvest storage, and they have a short storage time. In China, the postharvest loss of yellow peaches is as high as 20–30% [2]. To extend the storage time of yellow peaches to avoid yellow-peach waste is a problem and a challenge for the fruit industry. Over the years, researchers have investigated several different techniques to extend the postharvest life of yellow peaches, such as indirect plasma-treated air [1], humic acid treatment [3], H_2_S and NO [4], oxymatrine [5], 1-methylcyclopropene [6], and methyl jasmonate [7]. Although these preservation techniques can retard the fruit aging, their safety remains to be proven. Precooling allows fruits with high field heat to be cooled quickly, which effectively reduces the metabolic effects of the fruit and delays the multiplication of microorganisms, thus extending the storage time of the fruit, which is a safe treatment [8,9]. Therefore, it is necessary to study the effect of precooling on the storage time and quality changes of yellow peaches for the postharvest precooling treatment of yellow peaches.

Precooling is the process of removing field heat from freshly harvested agricultural products to reduce the metabolism and minimize spoilage before transportation or storage [10]. The level of field heat significantly affects the storage length of agricultural products. Studies have shown that, for most fresh agricultural products, a one-hour delay in precooling at field temperatures of 35 °C can reduce the storage time to about one day, even under optimal storage conditions [11]. Moreover, the rapid reduction in the fruit temperature through the precooling treatment allows the cooler to cool down more quickly to the ideal environmental temperature during the refrigeration process, and the fruit has a shorter time to reach the ideal temperature. These favorable conditions can extend the storage time of agricultural products and maintain the quality of the products. Methods of fruit precooling usually include RPC, CWPC, SWPC, FIPC, and VPC. These precooling methods have been used for a long time in the preservation of fruits and vegetables, such as asparagus, beans, cucumbers, sweet corn, blueberry, radishes, sweet cherries, peppers, lychee, and strawberries, and they have been proven effective [12,13].

Some other studies have reported the effect of different precooling methods on the storage life of peaches. Xuan et al. [14] found that forced-air precooling improved the storage quality and antioxidant properties of nectarines during storage and transportation. Pervitasari et al. [15] found that water-cooled and 1-methylcyclopropene-treated yellow peaches had reduced ethylene production during storage, maintained their hardness and soluble-solids content, and their shelf-life was extended. Caprioli et al. [16] found that cold-water precooling reduced the respiration-rate reduction and hardness loss, thereby extending the postharvest storage life of the product. Unfortunately, most of these studies explored the effect of one precooling method on the storage quality of yellow peaches, and could not select the best precooling method to extend the storage period of yellow peaches. It has been shown that different types of fruits and vegetables can be precooled in different ways; for example, lychee is very suitable for CWPC, while strawberries are prone to decay after water cooling [9]. Some products can be cooled by any precooling method without any quality loss; other products may be adversely affected by certain precooling technologies [17]. Therefore, it is necessary to select the appropriate precooling technique for yellow peaches to maintain the quality of the product.

In this study, RPC, CWPC, SWPC, FIPC, and VPC were used to precool freshly harvested yellow peaches. All the precooled yellow peaches were sealed in modified-atmosphere packaging (MAP) (12% CO_2_/4% O_2_/84% N_2_) and refrigerated at 4 °C for 15 days. Quality indicators (ethylene production, respiration rate, sensory characteristics, soluble solids, ascorbic acid, texture, titratable acid, degree of browning, weight loss) and the content of POD, PPO, PAL, SOD, and antioxidant enzymes were measured in yellow peaches. In these studies, we investigated the effect of different precooling methods on the quality and storage life of yellow peaches during refrigerated storage, and we also discussed the investigations into the methods of precooling and the mechanisms of the refrigerated transport of peaches by analyzing the changes in the quality indicators and enzyme content. To provide theoretical support for maintaing the quality of yellow peaches, we selected the appropriate precooling method for yellow peaches, and the mechanism of extending the storage life of yellow peaches by precooling treatment.

## 2. Results

### 2.1. Respiratory Rate and Ethylene Production

**Figure 1:**
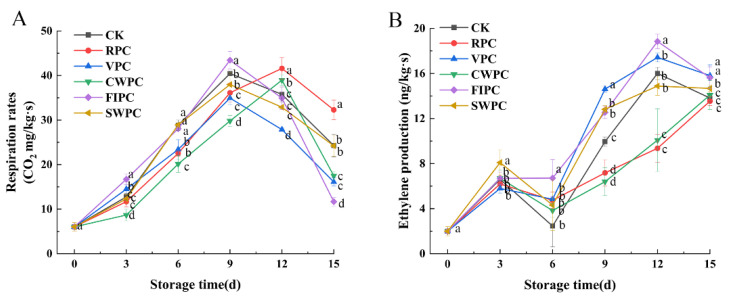
Changes in (**A**) respiration-rate and (**B**) ethylene production of yellow peaches under different precooling methods during cold storage. The abbreviations CK, RPC, CWPC, SWPC, FIPC, and VPC in the figure correspond to control group, refrigerator precooling, cold-water precooling, strong-wind precooling, fluidized-ice precooling, and vacuum precooling, respectively. Values with different lowercase letters in the same column are significantly different (*p* < 0.05).

The respiration rates of yellow peaches with different precooling methods during storage are shown in Figure 1A. From Figure 1A, it can be seen that the respiration rate of yellow peaches increased with the extension of the storage time, and a gradual decrease in the respiration rate after the peak of respiration appeared, which indicated that the yellow peaches had started to enter the aging stage. The peak respiration rates of the CWPC and RPC groups appeared on the 12th day, while the peak respiration rates of all the other treatment groups appeared on the 9th day.

Ethylene is a bioactive compound that is considered to be a naturally occurring hormone in plants. The variation in the ethylene production for different precooling treatments is shown in Figure 1B. As the ripening of menopausal fruit begins, ethylene production increases dramatically, which coincides with a rapid rise in respiration. Compared with the CK, the CWPC- and RPC-treated groups were able to delay the arrival of peak ethylene production, which occurred after day 15, while in the other treatment groups, peak ethylene occurred on day 12.

### 2.2. Sensory Evaluation, Browning, Weight-Loss Rate, and Texture

**Figure 2:**
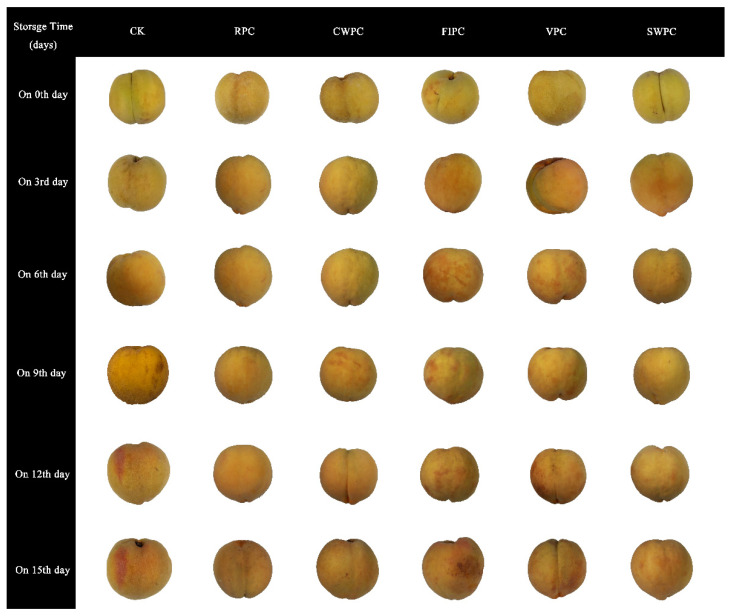
Appearance changes in yellow peaches with different precooling methods during cold storage. The abbreviations CK, RPC, CWPC, SWPC, FIPC, and VPC in the figure correspond to control group, refrigerator precooling, cold-water precooling, strong-wind precooling, fluidized-ice precooling, and vacuum precooling, respectively.

**Figure 3:**
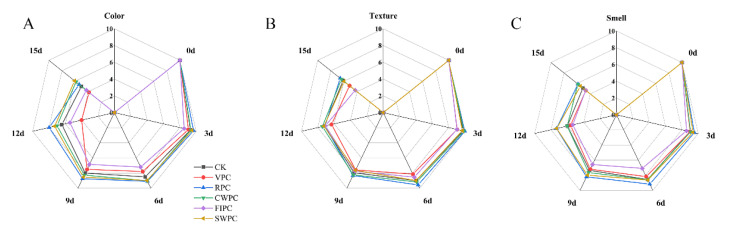
Sensory changes in yellow peaches with different precooling methods during cold storage: (**A**) color; (**B**) texture; (**C**) smell. The abbreviations CK, RPC, CWPC, SWPC, FIPC, and VPC in the figure correspond to control group, refrigerator precooling, cold-water precooling, strong-wind precooling, fluidized-ice precooling, and vacuum precooling, respectively.

Figure 2 and Figure 3, respectively, show the changes in the sensory photos and sensory scores of the yellow peaches at the sampling times during cold storage. It could be seen that the quality of all the yellow peaches deteriorated with the extension of time. After 15 days of storage, the sensory scores of the CWPC- and RPC-treated groups were higher than those of the other experimental groups. At the same time, the appearances of these two groups were obviously better than those of the other experimental groups. The sensory state of the VPC- and FPIC-treated groups during cold storage was poor, which was mainly due to the browning and wrinkling on the surfaces of the yellow peaches, which resulted in low scores for the appearance, color, and texture. The RPC- and CWPC-treated fruits aged slowly within 15 days, and they remained in a good sensory state at the end of storage (on the 15th day). The browning and folding of the SWPC-treated group were not serious during cold storage, and the sensory score was at a medium level during cold storage.

**Figure 4:**
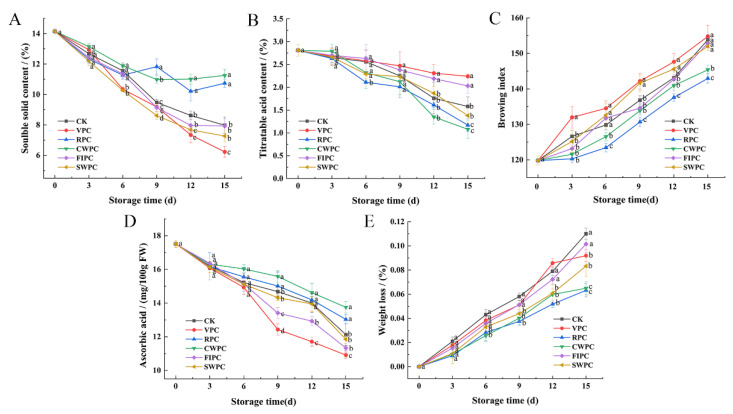
Changes in (**A**) soluble-solids content; (**B**) titratable acid content; (**C**) pericarp browning index; (**D**) vitamin C content; (**E**) weight loss of yellow peaches with different precooling methods during cold storage. The abbreviations CK, RPC, CWPC, SWPC, FIPC, and VPC in the figure correspond to control group, refrigerator precooling, cold-water precooling, strong-wind precooling, fluidized-ice precooling, and vacuum precooling, respectively. Values with different lowercase letters in the same column are significantly different (*p* < 0.05).

Browning is an important factor that leads to the quality deterioration of the postharvest storage quality of yellow peaches, and precooling treatment could mitigate this phenomenon. Browning occurred in all the samples during cold storage (Figure 4C). The browning of the VPC-treated samples began from day 3, and they had a significantly higher browning degree than the other samples at the end of storage. Compared with the CK, the browning of yellow peaches was less in the CWPC- and RPC-treated samples, indicating that these two precooling methods could inhibit the browning of yellow peaches in cold storage. The VPC- and FIPC-treated samples showed obvious browning on the 12th day and decayed at the end of storage (Figure 4).

Table 1 presents the texture results on day 0 and on the 15th day. The treatment of yellow peaches with different precooling methods resulted in different texture changes of the yellow peaches during cold storage (Table 1). The hardness, cohesion, and chewability of each treatment group showed a downward trend, while the viscosity showed an upward trend. The hardness of the yellow peaches in the different treatment groups decreased with the decrease in the cohesion. The cohesion of yellow peaches may be affected by their water content and soluble substances [18]. The water content and solid content of the yellow peaches treated with RPC and CWPC were higher than those of the other treatment groups, and so their cohesion was also higher than that of the other treatment groups. Compared with the other treatment groups, the RPC- and CWPC-treated samples stored for 15 days still had higher hardness, cohesion, and chewability. Therefore, the two groups of yellow peaches treated by the precooling methods had higher sensory scores and quality.

The loss of moisture during storage is the main reason for the weight loss [19]. The hardness of the CWPC- and RPC-treated yellow peaches was significantly higher than that of the other samples on the 15th day (Table 1, *p* < 0.05). Figure 4E also shows that the water-loss rates of these two groups were the lowest on the 15th day. At the end of storage, the weight-loss rate of the CWPC-treated samples was only 0.0649%, while the weight-loss rate of the FIPC-treated samples was as high as 0.1015% (Figure 4E).

### 2.3. Analysis by LF-NMR, Soluble-Solids Content, Titratable Acid Content, and Ascorbic Acid

**Figure 5:**
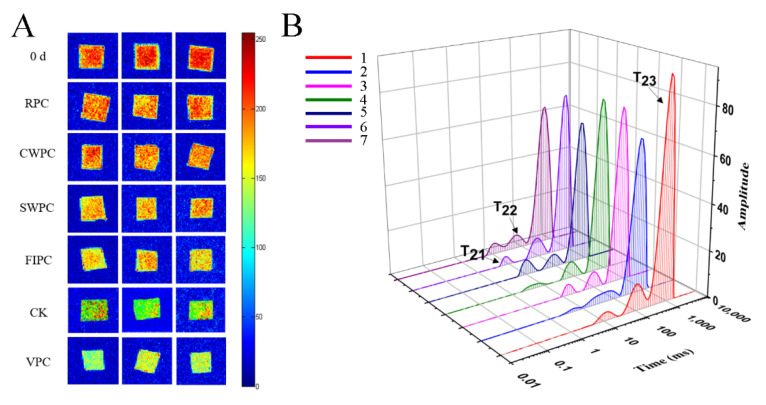
Analysis by LF-NMR: (**A**) plot of magnetic-resonance imaging on day 0 and day 15, where red is the region of high proton density, and blue is the region of low proton density; (**B**) plot of water distribution of yellow-peach samples on day 0 and day 15, where T_21_ represents bound water, T_22_ represents nonfluid water, and T_23_ represents free water; the peak and peak area represent the contents of different kinds of water; 1: Day 0; 2: CK; 3: RPC; 4: CWPC; 5: FIPC; 6: SWPC; 7: VPC. The abbreviations CK, RPC, CWPC, SWPC, FIPC, and VPC in the figure correspond to control group, refrigerator precooling, cold-water precooling, strong-wind precooling, fluidized-ice precooling, and vacuum precooling, respectively.

Water, which provides a medium for metabolic activity and maintains the fruit morphology, plays an important role during postharvest storage [20]. Low-field nuclear magnetic resonance (LF-NMR) techniques can precisely detect the distinct qualities and content of water based on the difference in the hydrogen-atom relaxation times in a magnetic field [21]. Magnetic resonance imaging (MRI) (Figure 5A) provides the visual information of yellow peaches during cold storage. Red was the region with high proton density, and blue was the region with low proton density [22]. The change in the water distribution of yellow peaches with the storage time is shown in Figure 5A. It can be seen from Figure 5A that the moisture content of the pulp is the highest. During the cold storage, the moisture content of the pulp decreases (red or yellow disappears). Moreover, the color of the samples treated with VPC was bluer than that of the other samples, which indicated that the VPC treatment caused more severe water loss. However, the samples treated with CWPC and RPC were redder, indicating less water loss. Three peaks correspond to the three relaxation components: T_21_ (<10 ms), T_22_ (20–400 ms), and T_23_ (>1000 ms), which represent bound water, fixed water, and free water, respectively [23]. The free water (T_23_) of the yellow peaches treated with CWPC and RPC decreased the least (Figure 5B), and the free-water content of the yellow peaches treated with VPC decreased the most. Immobilized water (T_22_) reflected the metabolic-reaction activity of the yellow peaches during cold storage. It could be seen that the immobilized water of the yellow peaches with different precooling methods was reduced compared with that on day 0, but the difference was very small, which could be due to the low temperature during cold storage that reduced the metabolic activity of the yellow peaches.

The content of soluble solids reflects the physiological status and quality of yellow peaches [24]. The soluble-solids content of the yellow peaches with different precooling methods decreased during cold storage (Figure 4A). After 15 days of cold storage, the soluble-solids contents of the yellow peaches treated by RPC and CWPC were higher (10.74% and 11.25%, respectively). However, the soluble-solids content of the VPC-treated peaches was the lowest, which decreased by 7.92% within 15 days.

Titrable acid contains a variety of organic acids, which are the substrate of fruit respiration and metabolism, and their contents will also affect the flavor of the fruit. The titratable acid content of yellow peaches treated by different precooling methods decreased slowly from day 0 to day 9, and then decreased significantly (*p* < 0.05). During the whole storage process, the curve of the titratable acid content of the VPC- and FIPC-treated samples was relatively gentle. After 15 days of cold storage, the titratable acid contents of the two precooling groups were 1.16% and 0.94% higher, respectively, than those of the CWPC-treated samples.

The ascorbic acid contents of yellow peaches treated with different precooling treatments decreased during cold storage (Figure 4D). The ascorbic acid contents of the RPC- and CWPC-treated groups were significantly higher than those of the other three samples from day 9 (*p* < 0.05), indicating that the CWPC- and RPC-treated samples still maintained high antioxidant activity at that time. The ascorbic acid contents of the CWPC- and RPC-treated peaches were higher, reaching 13.75 and 13.03 μg mL^−1^, respectively, at the end of storage. However, the contents of ascorbic acid in the SWPC-, FIPC-, VPC-, and CK-treated peaches were 11.85, 11.33, 10.91, and 12.12 μg mL^−1^, respectively.

### 2.4. Antioxidant-Related Enzyme Analysis

**Figure 6:**
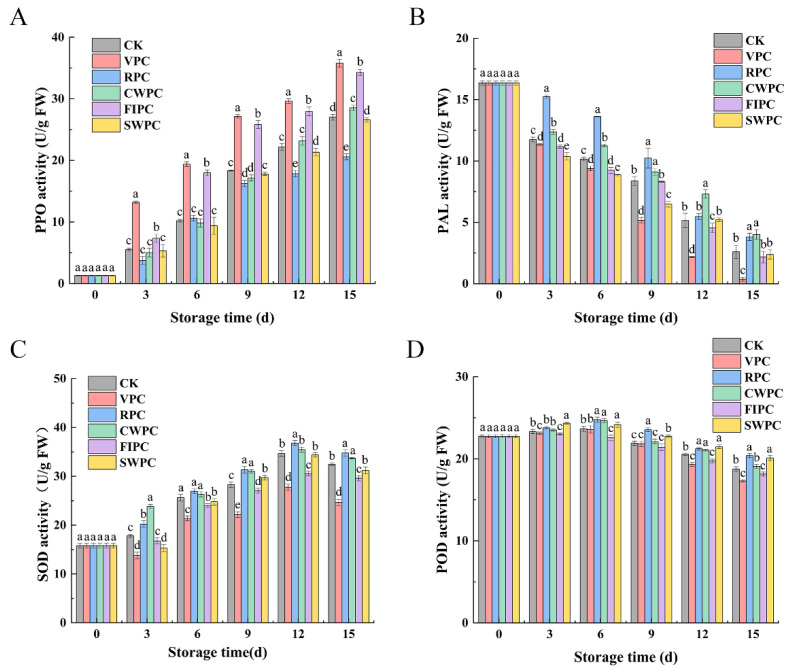
Changes in (**A**) PPO, (**B**) PAL, (**C**) SOD, and (**D**) POD activities of yellow peaches with different precooling methods during cold storage. The abbreviations CK, RPC, CWPC, SWPC, FIPC, and VPC in the figure correspond to control group, refrigerator precooling, cold-water precooling, strong-wind precooling, fluidized-ice precooling, and vacuum precooling, respectively. Values with different lowercase letters in the same column are significantly different (*p* < 0.05).

The PPO activity of all the samples increased during cold storage (Figure 6A). After 15 days of cold storage, the PPO activity of the RPC-treated samples was significantly lower than that of the other experimental groups (*p* < 0.05), which was 6.42 U/g FW lower than the CK. However, the PPO activity of the VPC-treated samples was significantly higher than that of the other samples (*p* < 0.05), which was 8.77 U/g FW higher than the CK.

The change in the PAL enzyme content is shown in Figure 6B. The PAL activity of the yellow peaches treated with different precooling methods decreased continuously during storage. However, the RPC- and CWPC-treated peaches showed higher PAL activity during storage. In contrast, the PAL activity of the yellow peaches treated by VPC was the lowest in the same period.

The activities of the SOD of the samples treated with RPC and CWPC were significantly higher than those of the other samples (Figure 6C, *p* < 0.05). However, the samples treated with VPC and FIPC showed the worst performance. After 15 days of storage, their contents were 7.85 and 2.89 lower than the CK, respectively.

The POD activity of the yellow peaches treated by different precooling methods increased at the early stage of storage (Figure 6D), and then decreased after reaching the peak. During storage, the POD activity of each precooling group did not change significantly, but after 15 days of refrigeration, the POD activity of the VPC- and FIPC-treated groups was significantly lower than that of the other groups in the same period. Compared with day 0, the POD activities of the VPC- and FIPC-treated groups decreased by 5.43 U/g FW and 4.60 U/g FW, respectively.

## 3. Discussion

### 3.1. Effects of Different Precooling Methods on Ethylene Production and Respiration Rate of Yellow Peaches

Yellow peaches are a menopausal fruit that exhibit a dramatic increase in the endogenous ethylene production and respiration rate during ripening [25]. Respiration is a metabolic process that provides energy for plant biochemical processes and accelerates plant maturation and senescence. Therefore, the inhibition of the fruit respiration rate can maintain the postharvest fruit quality and delay fruit senescence [26]. As seen in Figure 1, the respiration rate of the FIPC-treated samples remained at a high level during storage and had poor sensory scores and appearances (Figure 1, Figure 2 and Figure 3). The reason for this may be that the substrates consumed by respiration cause irrecoverable changes in the nutrition, taste, and appearance of the fruit [27]. CWPC and RPC not only delayed the onset of the peak respiration rates in the yellow peaches, but also inhibited the respiration rates to maintain the lower physiological and metabolic activities during storage, thereby delaying fruit senescence. Alique et al. [28] also found that water cooling reduced sweet-cherry respiration and the consumption of respiratory substrates, delaying softening and extending their shelf life.

Ethylene is important for the initiation and completion of ripening, and for the softening of menopausal fruits, such as peaches [29]. Therefore, reducing ethylene production can extend the storage life of fruits. Ethylene triggers the ripening process in menopausal fruits by stimulating endogenous ethylene biosynthesis and fruit softening [4]. In contrast, CWPC and RPC can delay the arrival of respiration and peak acetylene during yellow-peach storage and slow down the physiological metabolic changes, thus maintaining the quality of yellow peaches and extending their storage time.

### 3.2. Effect of Different Precooling Methods on the Postharvest Quality of Yellow Peaches

The cohesion of yellow peaches may be affected by their water content and soluble substances [18]. The water content and solid content of the yellow peaches treated with RPC and CWPC were higher than those of the other treatment groups, and so their cohesion was also higher than that of the other treatment groups. High cohesion maintains the product integrity during cold-storage operations, reduces water loss throughout the product shelf life, and maintains the maximum flavor, texture, and color [30]. Therefore, the sensory scores and quality of the yellow peaches were higher in both groups treated with CWPC and RPC. As one of the most important quality parameters, firmness determines the shelf life and consumer acceptance of fruits [31]. Fruit firmness usually decreases with ripening and aging [24]. The reason for maintaining higher firmness during the storage of CWPC and RPC may be that these two precooling methods delay the arrival of peak ethylene and reduce the respiration rate, thus delaying the ripening of yellow peaches. Li et al. also found that increased respiration and ethylene production resulted in accelerated ripening and the excessive softening of the peach flesh [32].

In addition, water loss is a major factor that contributes to the decrease in fruit hardness [33,34]. The present experimental study showed similar results. The CWPC-treated peaches had both high hardness and low water loss. This is probably because CWPC can reduce water loss, maintain the swelling pressure and elasticity, and delay the softening of yellow peaches [35]. Oliveira et al. [12] showed that CWPC can delay the softening of cashews and slow down the weight, which is consistent with the results of the present experimental study. Mukama et al. [36] also found that the weight loss of pomegranate can be reduced by different precooling methods, thereby maintaining the pomegranate hardness.

Fruit softening is caused by the breakdown of insoluble protopectin into soluble pectin, or by increased membrane permeability due to cell division [37]. Water loss and the reduced swelling of cells may be key factors in the softening of nonwater prechilled fruit tissues [38]. The high water loss in nonwater prechilled yellow peaches accelerates aging and thus alters the membrane permeability, making them susceptible to softening. Zainal et al. [38] also showed that weight loss in non-hydrocooled rock melon was associated with a loss of cell swelling, which affects the cell-wall stiffness of the fruit-rind tissue, whereas hydrocooled rock melon could maintain the cell-wall structure of the fruit and preserve its quality during refrigeration. In the yellow peaches treated with SWPC and VPC, the water loss was more severe due to the accelerated metabolism during refrigeration (Figure 4E). This explains the possibility of the faster aging and deterioration of the SWPC- and VPC-treated samples in cold storage. For example, water loss accelerates changes in the membrane permeability, leading to accelerated water loss in fruits during cold storage [39]. Different precooling methods and storage times affect the quality loss of yellow peaches. VPC produces the least free-water content, peach shrinkage, and softening, and a higher weight-loss rate. This is because water evaporates more quickly from the fruit during VPC, and so the weight loss in VPC can be more severe than in other precooling methods [40,41]. The salt-solution medium used in FIPC may lead to changes in the osmotic pressure of yellow-peach epidermal cells due to its high osmotic pressure. Therefore, the yellow peaches after FIPC also exhibited a high rate of water loss in cold storage.

During cold storage, the contents of the titratable acid and soluble solids of the yellow peaches decreased continuously, which was consistent with the results of Raffo et al. on yellow peaches [35]. The decrease in the soluble-solids content might be attributed to respiration and sugar conversion [42]. The higher content of soluble solids in the yellow peaches treated with RPC and CWPC may be due to the fact that the two treatments slowed down the respiratory and metabolic activities of the yellow peaches [37]. The contents of the total soluble solids and the total acidity have been directly related to the taste and sensory quality of fruits [43]. The CWPC- and RPC-treated peaches had good sensory quality, which was closely related to their late-storage characteristics of a high content of soluble solids (mainly soluble sugar) and low acidity. The various organic acids that yellow peaches can consume during respiration cause a decrease in the titratable acid content [44]. Therefore, the acidity decreases with the increase in the maturity or the extension of the storage time [45]. In Hanif’s experiment, the titratable acid of papaya also showed a downward trend during the whole storage process [46]. Precooling will reduce the rate of ethylene production and ripening [47], and different precooling methods may affect the respiration rate and fruit ripening, and may thus present different acidities [3]. On the 15th day, the weight loss of the yellow peaches treated with CWPC and RPC was at a low level (Figure 4E), and the acidity of these two groups of yellow peaches was also low. This is because the content of water will affect the concentration of acid, resulting in different acidities [36].

As an essential elementary metabolite in plants, ascorbic acid acts as an antioxidant, enzyme cofactor, and cell-signaling regulator in many important physiological processes, including cell walls, phytohormone biosynthesis and secondary metabolites, stress resistance, and photoprotection [43]. High antioxidant activity significantly inhibited the browning reaction. Figure 4C shows that the browning degree of the CWPC- and RPC-treated samples were always kept at the lowest level. This might be because the RPC and CWPC could quickly reduce the yellow peaches to cold-storage temperatures to reduce the oxidative stress in the respiratory rate, delay the fruit ripening and senescence, and produce better-quality yellow peaches. In addition, CWPC reduced the diffusion of oxygen, thereby reducing the loss of ascorbic acid in the yellow peaches.

### 3.3. Effects of Different Precooling Methods on the Antioxidant-Enzyme System of Yellow Peaches

The senescence and oxidation reaction of fruit during cold storage are going on all the time, while the POD, SOD, PAL, and PPO in plants are the four enzymes closely related to the antioxidant reaction [48]. The activities of these four enzymes reflect the oxidative activity of yellow peaches during cold storage. The color characteristics of the fruit could be used to assess the ripeness and quality, and they are also an influential factor in consumer preferences [49]. Browning is an important factor that leads to the quality deterioration of the postharvest storage quality of yellow peaches, and precooling treatments could mitigate this phenomenon. The three key enzymes involved in fruit browning are PPO, POD, and PAL [50,51,52]. The main reason for peach browning is the change in the PPO and POD activities [53]. In particular, PPO could catalyze the conversion of phenolic compounds into quinones, resulting in fruit browning [54,55]. POD is the main enzyme that responds to pericarp browning by rapidly degrading phenols and anthocyanins [56]. Phenolic compounds are oxidized to quinones in an electro-phenolic reaction, and the quinones could be converted to brown polymeric pigments by regulating the activity of POD enzymes [57]. The end products of oxidized phenolic compounds can release a large number of monosaccharides from the plant cell wall, in which the participation of air oxygen is seriously involved in this process, resulting in a high browning rate in fresh fruits [58]. The PPO activities of the CWPC- and RPC-treated groups were lower than those of the other groups (Figure 6), which also explained the low browning degree of the yellow peaches in the two groups. In addition, CWPC could maintain the pericarp water content, stabilize the cell integrity, separate the browning substrates and enzymes, and delay the browning of yellow peaches [13]. Brackmann et al. [59] showed similar results, finding that ethylene stimulated the activities of POD and PPO, which, in turn, oxidized the phenolic compounds leached from the damaged epidermis, causing the fruit to darken. Therefore, it can be inferred that CWPC and RPC alter the enzyme activity by delaying peak ethylene and the respiration rate, which, in turn, affects the appearance quality of yellow peaches. Oliveira et al. also reported that CWPC could effectively reduce the POD activity, delay weight loss, and better protect the vitamin C and color of cashew apple [60].

POD and SOD can also scavenge excess ROS and protect cell membranes from damage, thus keeping the fruit fresh. Therefore, increasing the POD activity of yellow peaches during storage is conducive to improving the resistance of yellow peaches. The POD activities of the CWPC-, RPC-, and SWPC-treated groups were significantly higher than those of the other groups in the same period, indicating that these three precooling methods can effectively improve the POD activity of yellow peaches during storage and help reduce the browning phenomenon of yellow peaches.

SOD can scavenge superoxide anion radicals generated during the physiological activities of yellow peaches, thus reducing the oxidative effects of free radicals on the tissues and cells [61]. In addition, SOD is beneficial in maintaining the balance of hydrogen peroxide in the cell wall and enhancing the defense of the fruit against undesirable external factors [62]. The SOD and POD activities of the RPC- and CWPC-treated samples were significantly higher than the other sample groups, indicating that the RPC and CWPC treatments could better maintain the ability of yellow peaches to scavenge oxygen radicals and reduce the damage of free radicals on macromolecules [63]. These precooling treatments may have delayed the biochemical reactions in yellow peaches, thus delaying fruit aging [64].

PAL is a key enzyme in the metabolism of phenylpropane, and it has great physiological significance for plants. In particular, PAL can improve the plant resistance when plants are exposed to unfavorable environments [65,66]. However, showing higher PAL activity, the good quality of the RPC- and CWPC-treated yellow peaches during storage could be attributed to their higher PAL activity, which could improve the resistance of yellow peaches to adverse external environments. In contrast, the yellow peaches treated with VPC had the lowest PAL activity during the same period, indicating that VPC was not effective in increasing the PAL activity of yellow peaches during storage, and therefore, their quality was the worst.

## 4. Materials and Methods

### 4.1. Fruit Materials

Yellow peaches (*Amygdalus persica)* were picked from a field in Fengxian, Shanghai (30°91’77.95’’ N, 121°47’40.42’’ E), free of mechanical damage, lesions, and ripeness (mass: 0.253 kg ± 2.0 g; hardness: 3016 ± 2.5 N; soluble solids: 14 ± 0.25%; titratable acid: 2.7 ± 0.25%). The harvested yellow peaches were then transported to the laboratory within 1 h for experimental processing.

### 4.2. Precooling Mode

The following five kinds of precooling treatments were carried out:

(i) SWPC: The centers of two yellow peaches were evenly dispersed at a 20 cm separation on shallow uncovered trays of 1.5 × 1.5 m, and were kept in a forced-air freezer at 4 °C, with an air speed of 8 m/s, for 40 min;
(ii) CWPC: The yellow peaches were hydrocooled in 200 L rectangular plastic water tanks, and sodium hypochlorite (NaOCl) (5.65–6.00%; Fisher Scientific, Springfield, NJ) was added to water to produce a final concentration of 100 ± 2 or 200 ± 2 ppm active chlorine (HOCl). The pH values of the solutions were adjusted to 6.8 ± 0.05 using a 6 N HCl solution (Fisher Scientific, Springfield, NJ, United States of America). The yellow peaches were placed in sterilized cold water for 70 min, with the cold-water temperature always maintained at 4 °C at all times;
(iii) VPC: The yellow peaches were kept in a VPC machine (VAC-0.2 type, Shanghai, China) for 260 min. The vacuum-cooler preset temperature was 4 °C, the water-spraying volumes were at 3%, and the final pressure was 580 Pa;
(iv) RPC: The centers of two yellow peaches were evenly dispersed at a 20 cm separation in a shallow uncovered dish of 1.5 × 1.5 m, and were kept in a refrigerator at 4 °C for 130 min;
(v) FIPC: The yellow peaches were kept in an ice slurry (ranging from −0.5 °C to −1.5 °C) for 80 min;
(vi) CK: The yellow peaches without any precooling treatment after picking.

The precooled yellow peaches were sealed in modified-atmosphere bags (12% CO_2_/4% O_2_/84% N_2_) and stored at 4 °C with a relative humidity of 55–65% during storage. The time required for all the precooling treatments (the time required for the central temperature of the yellow peaches to reach 4 °C) and precooling parameters were measured by pre-experiments.

### 4.3. Sample Collecting

In this study, 540 uniformly sized yellow peaches were randomly selected and divided equally into 6 experimental groups. After 0, 3, 6, 9, 12, and 15 days of storage, the yellow peaches were removed from the cold storage and placed in a constant temperature and humidity incubator (LHS-100CA, Shanghai Yiheng Instruments Co., Ltd., Shanghai, China) at 25 °C and 55–65% RH for 120 min before indexing. Yellow-peach tissues were isolated from 4–8 mm below the skin around the fruit equator for the physiological- and biochemical-index determination; yellow-peach puree from each sample was packed individually in aluminum foil and was immediately frozen in liquid nitrogen and stored at −80 °C until the enzyme-index determination was performed.

### 4.4. Respiratory Rate and Ethylene Production

At each time point, 9 peach fruits were randomly selected from each group and were divided into 3 subgroups. After placing the intact 3 peaches in a sealed container (4 L) at 25 °C for 2 h, a 10 mL gas sample was removed from the headspace gas body using a gas-tight syringe, and it was used for the gas-index determination for three times.

The ethylene production was determined by gas chromatography, as described previously [67]. A total of 5 mL of gas was injected into a gas chromatograph (GC-9A, Shimadzu, Japan) equipped with a GDX-502 column and a flame ionization detector (FID). The column temperature was 70 °C, and the injection temperature was 120 °C. The carrier gas was N_2_ , and the flow rate was 40 mL min^−1^.

The respiration rate was measured by taking 1 mL of the gas sample with a respirometer (PBI-Dansensor, CheckPoint, Denmark) [5]. The results were expressed in mg kg^−1^ s^−1^.

### 4.5. Sensory Characteristics

Twelve people with extensive experience in fruit sensory evaluation were engaged to form an assessment team to reasonably rate the color, texture, and smell of each sample during the experiment. The full score of the three indicators is 10. The specific evaluation criteria are shown in Table 2.

### 4.6. Determination of Texture, Browning Index, and Weight-Loss Rate

Three measurements were taken from opposite sides of each of the three yellow peaches cooled to room temperature, and they were repeated three times. The texture changes in the yellow peaches were analyzed using a texture-analyzer (TA-XT Plus C, Stable Micro Systems Co. Ltd., Surrey, UK) SMS-P/2 cartridge. The operating parameters were the same as those of mango, and the puncture depth depended on the thickness of the peach stem.

The browning index refers to the experimental method of Palou et al. [68], and it was modified. The L *, a *, and b * values were measured using a colorimeter (CR-400, Konica Minolta, Tokyo, Japan). The L * value indicates brightness, the a * value indicates red/green, and the b * value indicates yellow/blue. The browning index was calculated according to Equations (1) and (2):

(1) $$BI=\frac{100\times \left(x-0.31\right)}{0.175}$$

(2) $$x=\frac{{a\;}^{*}+1.75{L\;}^{*}}{5.645{L\;}^{*}+a{\;}^{*}-3.012{b\;}^{*}}$$

The fruit weight was monitored with an electronic scale (LQ-C10002, Shenzhen Feiya weighing apparatus Co., Shenzhen, China). The weight-loss rate was calculated as the percentage of the fruit-weight decrease compared to the initial weight [69].

### 4.7. Determination of Analysis by LF-NMR, Water Migration, Titratable Acidity, Soluble Solids, and Ascorbic Acid

The moisture distribution was examined in a low-field NMR analyzer (MesoMR23-060H-I, Suzhou Niumag Analytical Instrument Corporation, Suzhou, Jiangsu, China), according to the description by Xie et al. The yellow-peach slices (1 × 1 × 1 cm) were put into the 10 mm NMR tube. T_1_, T_2_, and T_1_–T_2_ experiments were performed in a 0.367 T (15.635 MHz) system (Spin Core Inc., Gainesville, FL, USA) with a 10 mm r. f. coil. Spin–lattice-relaxation (T_1_) measurements were performed using an inversion-recovery pulse sequence, with a delay time changing from 0.5 ms to 4.61 s, with 512 acquisition points and 4 scans. For the T_2_ measurements, a Carr–Purcell–Meiboom–Gill (CPMG) pulse sequence was used, with an echo time (TE) of 4 ms, 4000 echoes, and 16 scans. The CYCLOPS phase-cycling routine was integrated into the sequences by Spin Core Inc. (Gainesville, FL, USA). Non-negative least squares (NNLS) was applied to the T_2_-decay curves to obtain the relaxation spectra. Two-dimensional T_1_–T_2_ experiments were performed with an Inversion Recovery–CPMG experiment [21].

The titratable acid was determined using the method of Bai et al. [69]. The titratable acidity (TA) (%) was assessed by the titration of the juice against sodium hydroxide, and it was expressed as the % of citric acid. The fruit-quality measurements were replicated thrice. 

The soluble solids were determined according to the method of Zhang et al. [70]. Thoroughly grind 5 g of sample, take a drop of supernatant, use a digital refractometer (PR32a, ATAGO, Japan) to measure and record the reading, and repeat three times.

The extract used for the ascorbic acid determination was obtained by mixing 6 g of either fresh or cooked sample with 20 mL of an extraction solution, which contained 30 g/L meta-phosphoric acid and 80 mL/L acetic acid in HPLC-grade water. The homogenized samples were thoroughly ground at 4 °C. The homogenate was centrifuged at 10,000× *g* for 20 min at 4 °C. The sample was further filtered through a 0.45 µm filter. The content of ascorbic acid in the yellow peaches was determined by HPLC, according to the method reported by Lafarga et al. [71]. The unit of the ascorbic acid content is mg/100 g FW.

### 4.8. Determination of Peroxidase (POD), Superoxide Dismutase (SOD), Phenylalanine Ammonia-Lyase (PAL), and Polyphenol Oxidase (PPO) Activities

The extractions of the PPO and POD were performed by homogenizing the sample (2.0 g) with ice-cold acetic acid–sodium acetate buffer (100 mmolL^−1^, pH 5.5) in a test tube. The mixture was centrifuged at 10,000× *g* at 4 °C for 20 min. According to the methods of Adhikari et al. and Lo’ay et al. [72,73], the supernatant was taken to determine the enzyme activity. For the PPO assay, the reaction system consisted of 4 mL of acetic acid–sodium acetate buffer (50 mmolL^−1^, pH 5.5), 1 mL of catechol solution (50 mmolL^−1^), and 0.1 mL of supernatant. One unit of PPO activity was represented as an increase in the OD_420_ of 1 per kilogram (fresh weight) per minute. For the POD assay, the reaction system consisted of 0.2 mL of H_2_O_2_ solution (50 mmolL^−1^), 3 mL of guaiacol solution (25 mmolL^−1^), and 0.5 mL supernatant. One unit of POD activity was represented as an increase of 1 in the OD_470_ per kilogram (fresh weight) per minute. The enzyme activities of the PPO and POD were expressed as U/g FW.

For the extraction of the enzymes, the sample was ground to powder in liquid nitrogen and homogenized in a prechilled mortar and pestle in 1.5 mL ice-cold extraction buffer containing 50 mM Na-phosphate buffer (pH 7.8), 1 mM ethylene diaminete traacetic acid (EDTA), and 1.0 percent (*w*/*v*) polyvinyl-pyrrolidone (PVP). The supernatant was used to determine the SOD activity. The superoxide dismutase (SOD) activity was assayed by measuring its capacity to reduce nitro-blue tetrazolium (NBT). The absorbance of the reaction solution was measured at 560 nm. One unit of SOD was defined as the enzyme activity that inhibited the reduction of nitroblue tetrazolium to blue formazan by 50 percent. The total SOD activity was expressed as U/g FW [70].

For the phenylalanine ammonia-lyase (PAL)-activity assay, 1.0 g of fresh yellow-peach fruit sample was homogenized with 5.0 mL of ice-cold sodium borate buffer (100 mM, pH 8.8) containing 5 mM β-mercaptoethanol, 2 mM ethylene diaminetetraacetic acid, and 4% (*w*/*v*) polyvinyl pyrrolidine. The homogenized sample was then thoroughly ground at 4 °C. The homogenate was centrifuged at 12,000× *g* for 30 min at 4 °C. The supernate was then collected for the enzymatic assay. The PAL activity was determined according to the method described by Shi et al. [74]. The specific enzyme activity was expressed as units (U) per gram of FW. One unit of PAL activity was defined as the amount of enzyme that caused an increase of 0.01 in the absorbance at 290 nm in 1 h under specified conditions.

### 4.9. Data Analysis

The data were analyzed by one-way ANOVA with IBM SPSS Statistics 25.0, and a Tukey test showed that the difference was statistically significant (*p* < 0.05). The results were expressed as means and standard deviations (SDs). The data statistics were performed by Microsoft Excel 2019, and the figure legends were drawn using Origin 2019b. All the experiments were repeated at least three times.

## 5. Conclusions

In this study, we investigated the effect of different precooling treatments (RPC, CWPC, FIPC, SWPC, and VPC) on the quality and storage life of the MAP (12% CO_2_/4% O_2_/84% N_2_) of yellow peaches stored at low temperatures (4 °C) for 15 days. It was confirmed that not all the precooling methods positively influenced the storage life of the yellow peaches, with the RPC and CWPC treatments showing better performances, while the FIPC, SWPC, and VPC treatments accelerated the deterioration of the yellow peaches. It was shown that the storage period of yellow peaches with RPC and CWPC treatments was 15 days, which was 6 days longer than that of VPC and FIPC. This is because the RPC and CWPC treatments delayed the peak of respiration and ethylene production, and increased the antioxidant-enzyme activity, thus maintaining the quality of the yellow peaches. In contrast, the VPC and SWPC treatments caused water loss in the yellow peaches, thus affecting the metabolism and antioxidant-enzyme activity, leading to the accelerated deterioration of the yellow peaches. The damage on the surfaces of the yellow peaches after the FIPC treatment may be due to the cold damage caused by too low temperatures acting locally on the yellow peaches, and the fluidized-ice treatment showed the worst storage level. In conclusion, the CWPC and RPC treatments are suitable precooling methods for the postharvest storage of yellow peaches to extend their storage shelf life.

## Figures and Tables

**Table 1:** Hardness, stickiness, elasticity, cohesiveness, and chewiness of yellow peaches on day 0 and the 15th day of storage.

	Hardness	Adhesiveness	Springiness	Cohesiveness	Chewiness
0d	3015.33 ± 848.92 ^a^	−10.28 ± 5.24 ^a^	0.52 ± 0.20 ^ab^	0.20 ± 0.02 ^a^	333.51 ± 183.75 ^a^
CK	855.3 ± 132.3 ^c^	−30.29 ± 13.14 ^ab^	0.60 ± 0.12 ^ab^	0.17 ± 0.03 ^a^	72.18 ± 12.12 ^b^
RPC	1527.22 ± 202.64 ^b^	−31.78 ± 15.21 ^ab^	0.58 ± 0.09 ^ab^	0.20 ± 0.03 ^a^	181.07 ± 64.69 ^b^
CWPC	1088.70 ± 114.18 ^bc^	−23.74 ± 14.23 ^ab^	0.43 ± 0.18 ^b^	0.17 ± 0.03 ^a^	75.89 ± 16.23 ^b^
SWPC	712.18 ± 121.31 ^c^	−31.42 ± 7.19 ^ab^	0.64 ± 0.05 ^ab^	0.17 ± 0.03 ^a^	73.17 ± 6.42 ^b^
FIPC	655.42 ± 38.13 ^c^	−36.72 ± 8.36 ^b^	0.76 ± 0.09 ^a^	0.16 ± 0.02 ^a^	78.50 ± 4.46 ^b^
VPC	500.80 ± 53.43 ^c^	−29.89 ± 17.76 ^ab^	0.70 ± 0.21 ^ab^	0.15 ± 0.03 ^a^	58.12 ± 29.73 ^b^

Note: The abbreviations CK, RPC, CWPC, SWPC, FIPC, and VPC in the figure correspond to control group, refrigerator precooling, cold-water precooling, strong-wind precooling, fluidized-ice precooling, and vacuum precooling, respectively. Results are expressed as means ± standard deviations (SDs). Values with different lowercase letters in the same column are significantly different (*p* < 0.05).

**Table 2:** Sensory-evaluation project.

Score	Color	Texture	Smell
10	Full and bright color	Crisp	Refreshing fragrance
8	Color is a bit dim	It is brittle, but it does not shrink.	No fragrance, no peculiar smell
6	Overall acceptable; color is a little dark with small spots.	Slight atrophy	No fragrance, slightly peculiar smell after careful smelling
4	Browning rate < 1/3	Obvious atrophy, but not serious.	Obvious odor, but not serious
2	Browning rate 1/3	Serious atrophy	Severe odor
0	All browning, and the color of mildew spots can be seen.	All severely atrophied and moldy.	Stench

## Data Availability

The data presented in this study are available upon request from the corresponding author.
